# Development and characterisation of a novel cell line derived from coho salmon (*Oncorhynchus kisutch*)

**DOI:** 10.1007/s11626-025-01113-3

**Published:** 2025-10-14

**Authors:** Mari Austad, Jacob Seilø Torgersen, Beate Beatriz Furevik, Lucy E. J. Lee, Guro Katrine Sandvik

**Affiliations:** 1 Centre for Integrative Genetics (CIGENE), Faculty of Biosciences, Norwegian University of Life Sciences (NMBU), Ås, Norway; 2 Department of Biology, University of the Fraser Valley, Abbotsford, British Columbia Canada; 3 AquaGen, Ås, Norway

**Keywords:** Fish cell line, *Oncorhynchus kisutch*, Coho salmon, In vitro

## Abstract

Coho salmon (*Oncorhynchus kisutch*) is an important salmonid species differing from other salmonids in its tolerance and response to pathogens endemic to the aquaculture industry, such as infectious pancreatic necrosis virus (IPNV) and infectious salmon anaemia virus (ISAV). Consequently, coho salmon has become a subject of increased scientific interest to investigate the underlying genetic mechanisms behind these and other host-pathogen interactions. Currently, most research studying coho salmon has been conducted using live animal models as there have been few in vitro tools readily available. Here, we present the first cell line from an adult coho salmon, Coho Salmon Fibroblast-Like 1 Norway-Canada (CSFL-1NC) and its preliminary characterisation. CSFL-1NC is a homogenous, spontaneously immortalised cell line from the pectoral fin of a wild adult coho salmon, with a consistent and stable fibroblastic morphology. The cell line has a relatively stable transcriptome across several passages, with high expression of key fibroblastic marker genes, displays rapid migration, and can be genetically manipulated both by transfection and transduction with varying efficiency using plasmids, lentivirus, and/or CRISPR methodology. Virus challenges show clear susceptibility to IPNV as evidenced by cytopathic effects and efficient viral replication, yet it shows little to no response when exposed to ISAV (HPRD).

**Supplementary Information:**

The online version contains supplementary material available at https://doi.org/10.1007/s11626-025-01113-3.

## Introduction

Coho salmon (*Oncorhynchus kisutch*), also known as silver salmon, is one of the twelve fish species belonging to the genus *Oncorhynchus* and one of the five salmonid species native to the North Pacific Ocean (Xuereb *et al*. 2022). Coho salmon holds substantial economic value, not only as a key fisheries and aquaculture species but also as a preferred species for many recreational fishermen (Auld *et al*. 2021; Xuereb *et al*. 2022). However, wild coho salmon stocks are in decline, leading to the species becoming increasingly threatened. Similarly to other salmonid fishes, this decline can be attributed to an array of factors including climate change and overfishing, as well as challenges endemic to a booming aquaculture industry (Larsen & Vormedal 2020; Mordecai *et al*. 2021; Xuereb *et al*. 2022).

Arguably, some of the biggest issues stemming from the aquaculture industry are the prevalence of viral diseases such as infectious pancreatic necrosis virus (IPNV), a non-enveloped, double-stranded RNA virus, and infectious salmon anaemia virus (ISAV), an enveloped, single-stranded RNA virus (Kibenge* et al*. 2001; Miller *et al*. 2014; Jorquera *et al.* 2016). While both are natural pathogens of fish, intensive farming of salmon in large densities can inflate transmission chances to unnatural levels, increasing infection pressure on both farmed and wild fish stocks (Jansen *et al.* 2012; Miller *et al.* 2014; Mordecai *et al*. 2021). The two viruses differ both in their modes of infection and their pathological symptoms, with IPNV primarily targeting the pancreas and liver of juvenile salmonids, leading to necrosis and high mortality, and ISAV primarily infecting endothelial cells, leading to haemorrhaging and anaemia in infected fish (Kibenge *et al*. 2001; Rolland & Winston 2003; Jorquera *et al. *2016; Duan *et al.* 2021). Interestingly, while these viral infections can be detrimental to Atlantic salmon farming, the presence of ISAV (HPRD) in Pacific salmonids does not cause clinical disease even at higher titres (Kibenge *et al*. 2001; Rolland & Winton 2003; Alarcon *et al*. 2020). However, this appears to be strain dependent, as some isolates may cause more significant clinical symptoms (Kibenge *et al.* 2001; Alarcon *et al*. 2020). Furthermore, while there is evidence that coho salmon can become infected with IPNV, there is little evidence supporting the same detrimental and deleterious effects seen in Atlantic salmon (Jorquera *et al*. 2016; Duan *et al.* 2021).

Traditionally, most studies investigating the characteristics of coho salmon have been done in vivo, using live animal models, due to a distinct lack of suitable in vitro models. These studies often involve challenge experiments where fish are deliberately exposed to viral agents followed by euthanasia of the fish or sampling of areas of interest for further analysis (Miller *et al*. 2014; Larsen & Vormedal 2020). This approach is both time consuming and costly and raises significant ethical concerns for the infected fish as it is in direct conflict with the three R’s of animal research: replacement, reduction, refinement (Fent 2001, Goswami *et al*. 2022; Nguyen *et al*. 2024). Therefore, developing in vitro models and tools, such as relevant cell lines, to help further our understanding of the complexity of the coho salmon immune system and response to viral pathogens has become increasingly important.

Both primary and established cell lines are valuable tools in in vitro biology, offering a complementary approach to in vivo studies. In vitro models both reduce the need for live animals while also offering a simplified model system with diminished “background” noise for enhanced reproducibility and easier control of experimental conditions (Fent 2001; Geraghty *et al*. 2014). However, it is essential to use in vitro studies in conjunction with and as a complement to in vivo studies to fully understand the complex physiological and immunological responses within an organism when studying complex processes such as viral infections and immune responses. Integrating both methods, rather than treating them as separate, typically yields the most robust and reliable scientific evidence. A variety of cell lines from a plethora of animals already exist, with Cellosaurus, the online resource for tracking cell lines, currently reporting the existence of over 163,868 unique cell lines (Bairoch 2018). Amongst these, there are already several cell lines from teleost fish, many of which have been particularly useful in investigating the toxicity of environmental contaminants on aquatic health; however, the only logged cell lines from coho salmon originate from embryos and are not readily available (Fent 2001; Bairoch 2018; Goswami *et al.* 2022).

Regardless of the species of origin, the characteristics of what makes a cell line a good tool for research are largely identical. In common for many of the most used cell lines are key characteristics such as stability of gene expression and phenotypically across passages, permissiveness to genetic editing by different methods, and demonstrable reproducible results (Geraghty *et al*. 2014). Additionally, the ease with which a cell line can be maintained in the laboratory, coupled with its availability, certainly affects its perceived scientific quality and, by extension, its usage in research (Bols *et al*. 2023).

Fibroblasts are a group of heterogenous cells present in virtually all tissues and organs of most animal species. Although heterogenous as a group, they do share some key marker genes and similar features (Plikus *et al*. 2021; Parker *et al*. 2023). They are often found within connective tissue where their main function is to secrete factors such as collagen, elastin, and fibronectin to support the extracellular matrix, all of which are essential to maintain essential organ functions (Plikus *et al*. 2021; Talbott *et al*. 2022; Knoedler *et al.* 2023; Parker *et al*. 2023). Although involved in many biological processes, they have for a long time been considered immunologically inactive and as such excluded from immunological research. However, there appears to be some immunological function within them, as fibroblasts from mammals, vertebrates, and zebrafish have been shown to express cytokines, important signalling proteins during inflammation (Ingerslev *et al.* 2010; Kendall & Feghali-Bostwick 2014). Furthermore, it has been suggested that fibroblasts could be viewed as sentinel cells within immune responses, activating and regulating gene expression post pathological stimuli, as they participate heavily in wound healing where they react to signals from adjacent wounded cells and appear to participate in inflammation; however, as they are a diverse group, this may not be applicable for all fibroblastic cells (Ingerslev *et al*. 2010; Kendall Feghali-Bostwick 2014; Talbott *et al. *2022).

Here, we present, to the authors’ best knowledge, the establishment and characterisation of the first cell line, Coho Salmon Fibroblast-Like 1 Norway-Canada (CSFL-1NC), from an adult coho salmon specimen. We show here a preliminary characterisation of its maintenance, morphology, and transcriptomic profile, as well as its migratory capabilities, response to two common salmon viruses, and permissiveness to gene editing via different methodologies.

## Materials and methods

### Sample collection

Offal of freshly caught wild adult coho salmon were donated by local recreational fishermen in the Fraser Valley, Abbotsford, Canada.

### Development of primary culture

Samples were rinsed in tap water at ambient temperature and carefully sprayed with a 70% ethanol (EtOH) solution. Relevant tissues (e.g. skin, fins, scales, gills) were excised and transferred to sterile petri dishes containing 1× Hanks’ balanced salt solution (HBSS). Excised fragments were then manually processed by scalpel and tweezers into smaller fragments of approximately 2.5 mm in size in droplets of 1× TrypLE™ (ThermoFisher, Waltman, MA). Explants were placed into 12.5 cm^2^ no-filter cap cell culture flasks (Corning Incorporated, Corning, NY) containing initial growth medium: Leibovitz’s L-15 medium with GlutaMAX™ (L-15, ThermoFisher) supplemented with 15% foetal bovine serum (FBS, Sigma-Aldrich, St. Louis, MO), 1% penicillin-streptomycin (Pen-Strep, 100 μg/mL, Gibco, Grand Island, NY), and 1% amphotericin B (2.5 μg/mL, ThermoFisher). Flasks were incubated in a 20 °C normoxic incubator overnight.

Following, flasks were observed daily to assess for attached explants, cell migration, and proliferation, as well as signs of contamination (i.e. bacteria, fungi, yeast). Several flasks yielded continuously dividing cells; however, the original flask of CSFL-1NC (from the pectoral fin of an adult wild specimen) displayed the greatest potential and, as such, was chosen for further work. Upon confluency, cells were washed twice with HBSS and detached using 1× TrypLE™ before being moved to a larger flask. Once confluent within this flask, the cells were sub-cultured at a 1:2 ratio as described below.

### Cell maintenance

Following the initial subculture, cells were routinely given new medium every 2–3 d and maintained in T75 cm^2^ no-filter cap cell culture flasks (Corning Incorporated) in a 20 °C normoxic incubator. When changing medium, half of the original medium was filtered through a 0.2-μm filter (Corning Incorporated) and stored at 4 °C for later use as conditioned medium. Once confluent, cells were rinsed two times with 1× PBS (Sigma) and detached using 2–3 mL of 1× TrypLE™. Cells are resuspended in 10 mL of growth medium (L-15 supplemented with 20% FBS and 1% Pen-Strep) and half moved to a new flask, accounting for a new passage. The cell line has routinely been tested for mycoplasma infection using the Mycostrip™ – Mycoplasma Detection Kit (InvivoGen, San Diego, CA) according to the manufacturer’s protocol.

Samples of CSFL-1NC have been routinely frozen at different passages to create a biobank of the cell line. Once 80% > confluent, cells have been detached as described above. Following, they have been centrifuged at 450 G for 5 min, and the resulting pellet resuspended in 0.5 mL FBS and transferred to 1.8 Nunc™ Biobanking and Cell Culture Cryogenic Tubes (Thermo Scientific). Cold freezing medium consisting of 4 parts growth medium and 1 part 20% dimethyl sulfoxide (DMSO, Sigma) is slowly added, and vials placed in a Mr. Frosty™ Freezing Container (ThermoFisher) for 2–3 d at −80°C. Finally, vials have been stored at −150°C for long-term storage. One confluent T75 flask yields two cryogenic tubes of cells for freezing. Frozen cells have been thawed by removal from the freezer and thawing at room temperature before centrifugation at 450 G for 5 min to remove DMSO, and the resulting pellet resuspended in growth medium and re-seeded in T25 flasks.

### Proliferation and doubling time

To identify and characterise the growth of the cell line, 5000 cells from P15 were seeded per well in 96-well cell culture CELLSTAR plates (VWR, Radnor, PA) and grown as described previously (“Cell maintenance” section). Every 24 h, cell viability was measured using the CellTiter-Glo™ Luminescent Cell Viability Assay (Promega Corporation, Madison, WI) according to the manufacturer’s protocol.

Luminescence readings were converted to estimated cell numbers using a standard curve generated from known cell dilutions. Mean cell numbers and standard errors were calculated across biological replicates (*n* = 4). Growth rates were calculated as the first derivative of the natural logarithm of mean cell number with respect to time (i.e. Δln(cell number)/Δtime), and doubling times were calculated as ln(2) divided by growth rate. The exponential growth phase (days 2–6) was determined by visual inspection of log-transformed growth curves.

### Morphology and immunostaining

To best visualise cell morphology and investigate cell identity, cells at passage 23 were seeded in 35-mm dishes (Sarstedt, Nümbrecht, Germany) and grown to semi-confluency. They were stained with Calcein-AM (1 μM, Sigma) and visualised by light and fluorescence microscopy (EVOS M5000 Imaging System, ThermoFisher).

Additionally, cells at passage 28 were stained with an antibody against alpha smooth muscle actin (α-SMA, GTX100034, GeneTex, Irvine, CA) as a marker for activated fibroblasts (Nielsen *et al.* 2019). First, cells were seeded onto a 4-well slide (IBDI) and grown to semi-confluency. Once semiconfluent, they were washed twice in 1× PBS and fixed in 2% paraformaldehyde (PFA) in 1× PBS for 15 min at room temperature. Following this, cells were washed in 1× tris-buffered saline with 0.1% Tween (TBST) two times for 5 min each. Cells were permeabilised for 15 min in TBST with 0.25% Triton X-100 and re-washed twice with TBST for 5 min. Cells were incubated for 60 min in blocking solution consisting of TBST with 0.02% Triton X-100 and 2% dry skimmed milk powder before being stained with 5 μg of the α-SMA antibody overnight at 4 °C in blocking solution. On the second day, cells were washed five times for 10 min each in TBST. Cells were incubated in the dark for 1 h with an anti-rabbit Alexa Fluor Plus 555 secondary antibody (A32732, ThermoFisher) at a 250× dilution with the inclusion of 4′,6-diamidino-2-phenylindole (DAPI nucleic acid stain, ThermoFisher) counterstaining at 1 μg/mL in the first wash step.

### Cell migration

To assess the cell lines’ migratory capabilities, cells at passage 18 were seeded in 12-well plates (ThermoFisher) and grown to confluency. To induce an artificial wound, a P20 pipette tip was used by dragging it through the cell monolayer in one swift downward motion from the top of the well to the bottom. Cell medium was changed from regular growth medium with 20% FBS to one containing only 2% FBS to prevent cell proliferation.

### Flow cytometer

In order to assess and ensure the homogeneity of the cell line, cells from passages 12 and 15 were sorted based on size using a SH800 cell sorter (Sony, Tokyo, Japan) according to the manufacturer’s protocol. Cells were re-seeded into a six well-based plate according to size and monitored while grown to confluency to determine any morphological differences between groups.

### RNA sequencing and transcriptomic analysis

To investigate the CSFL-1NC transcriptome, RNA samples from passages 11, 20, and 26 were sent for RNA sequencing. Total RNA was isolated from samples using the RNeasy Mini Kit (Qiagen, Venlo, Netherlands) according to the manufacturer’s protocol. Quality control and sample concentration were measured using a TapeStation 4150 (Agilent) and samples passing in-house quality control (RNA integrity number (i.e. RIN > 7) sent to a commercial provider (Novogen, Cambridge, UK). The generated data has been deposited in the European Nucleotide Archive (ENA) under accession number PRJEB90578.

Sequencing results were processed using the nf-core/RNASeq pipeline (Patel *et al*. 2024) to both ensure adequate quality and to quantify gene expression. Transcript per million (TPM) gene counts were used for further analysis. Any genes expressed below a set threshold of 1 TPM were filtered out before further analysis. The top 1000 genes expressed per passage were identified, as well as any overlap between passages. Gene ontology (GO) enrichment analysis was conducted to identify the biological function of genes and to identify groups of genes with similar expression patterns across passages; clustering was performed using self-organising maps (SOMs) in R. The number of clusters was selected based on exploratory testing, and the SOM algorithm was trained using default parameters with Euclidean distance metrics. Additionally, key fibroblast markers were identified from the literature, such as collagen variants, fibronectin, and more, and their expression was tracked across passages.

### Susceptibility to common salmon viruses

The cells were tested for their susceptibility to two common salmon viruses at passage 36: infectious pancreatic necrotic virus (IPNV, SP strain, isolate V-1244) and infectious salmon anaemia virus (ISAV, isolate described in Løkka *et al.* 2023) using viral titres of 7.5 × 10^8^ and 5.62 × 10^8^ TCID50/mL, respectively. Viral titres and MOI values (0.1 and 0.5) were selected based on prior reports of infections in salmonid cell lines, as well as unpublished optimisation experiments conducted in our laboratory on related salmonid cell lines (Weli *et al.* 2013; Løkka *et al*. 2023).

Seventy thousand cells were seeded per well in one 24-well plate per virus. Each plate had samples in triplicates of each condition, which were inoculated with their respective viruses at a multiplicity of infection (MOI) of either 0.1 or 0.5 for 1 h at 15 °C before the cells were washed in 1× PBS and the medium changed to growth medium. In the following days, the cells were visually inspected for any cytopathic effects, with images taken on day 5.

Post-infection, cells incubated with 0.5 MOI were detached following normal protocol on day 5 and total RNA isolated using the RNeasy Mini Kit (Qiagen). The concentration and quality control of the subsequent samples were conducted using a NanoDrop™ 8000 spectrophotometer (Thermo Scientific). qPCR was performed using a one-step qPCR iTaq Universal SYBR Green (Bio-Rad, Hercules, CA). The primers used for detection of viral replication were previously validated (Benkaroun *et al*. 2021; Kibenge *et al*. 2000) (see supplementary dataset 1 for primer sequences). Viral load was estimated based on the virus RNA level, normalised to the RNA level of a stably expressed gene in coho cells. This was calculated by the 2^−ΔΔCT^ method, where CT is the threshold cycle where the fluorescence in the qPCR reaction accumulates to a certain amount defined as the threshold (Livak & Schmittgen 2001).

### Transduction

The cell line transduction potential was tested using a lentivirus system with a green fluorescent protein (GFP) reporter gene. Lentiviral reporter particles were generated by transfecting packaging ps-PAX2 (#12260 Addgene, Watertown, MA), pMD2.G envelope ( #12259, Addgene), and transfer pLenti CMV GFP Puro (#17448, Addgene) plasmids into HEK293 cells as described previously (Humagain *et al.* 2024). One hundred thousand cells from passage 38 were seeded per well in a 24-well plate. On the day of transduction, cells were inoculated with lentivirus at 10 MOI overnight at 25 °C to ensure optimal infection conditions. The following day, the medium was changed and cells washed with 1× PBS. Cells were incubated with growth medium at 20 °C days before with puromycin (Sigma-Aldrich) selection. GFP activity was monitored using fluorescence microscopy (Olympus Scientific Solutions, Waltham, MA) to assess for GFP-positive cells.

### Transfection

The Neon™ Transfection System (ThermoFisher) was used to assess the cells’ permissiveness to transfection. On the day of transfection, cells from passage 30 were harvested as previously described and counted using the Countess™ 3 FL Automated Cell Counter (Invitrogen, Waltham, MA). One hundred and twenty thousand cells were used per reaction in a 10-μL reaction, each reaction accounting for one well in a 24-well plate. The pTagGFP2-N plasmid (Evrogen, Moscow, Russia) encoding the Enhanced Green Fluorescence Protein (eGFP) was used at a range of 0.7–1.2 μg per reaction. Briefly, post counting, the cells were washed once in HBSS with no Ca^2+^ or Mg^2+^, spun down at 450 G, and resuspended in buffer R before electroporation following the manufacturer’s protocol using the following settings: 1400 V, 2 pulses, 20 ms. Post electroporation, cells were incubated overnight in growth medium with no antibiotics. The next day, the medium was changed to normal growth medium with antibiotics.

### Gene editing by CRISPR-Cas9 knock out

To test permissiveness of the cells to gene editing using the CRISPR-Cas9 system, different knock out (KO) guides targeting *itgb4* and *itgb4-like*. This gene was selected as a target due to its high and consistent expression across passages, providing a robust target for editing efficiency, and due to its relevance for other projects within our laboratory. Guide-RNAs (gRNAs) and primers were designed in CHOPCHOP with the Okis_V2 assembly (GCF_002021735.2) version of the coho genome (see supplementary dataset 2 for sequences). Ribonucleoprotein (RNP) complexes were assembled as described in the Alt-R CRISPR-Cas9 System Protocol from Integrated DNA Technologies (Integrated DNA Technologies, INC 2019) and cells at passage 31 were electroporated as described above with 2.0 × 10^5 cells per well in a 12-well plate.

To assess guide efficiency, total DNA was extracted from the cells using a DNA Blood & Tissue Kit (Qiagen) according to the manufacturer’s protocol. Samples were measured and quality controlled on a NanoDrop™ 8000 spectrophotometer and amplified by PCR using DreamTaq Polymerase (ThermoFisher). Samples were cleaned using the ExoSap-IT PCR Product Clean Up Reagent Kit (ThermoFisher) and sent for sequencing at Eurofins Genomics. Results were analysed using the ICE CRISPR Analysis Tool at Synthego.

## Results

### Establishment of CSFL-1NC

**Figure 1.**
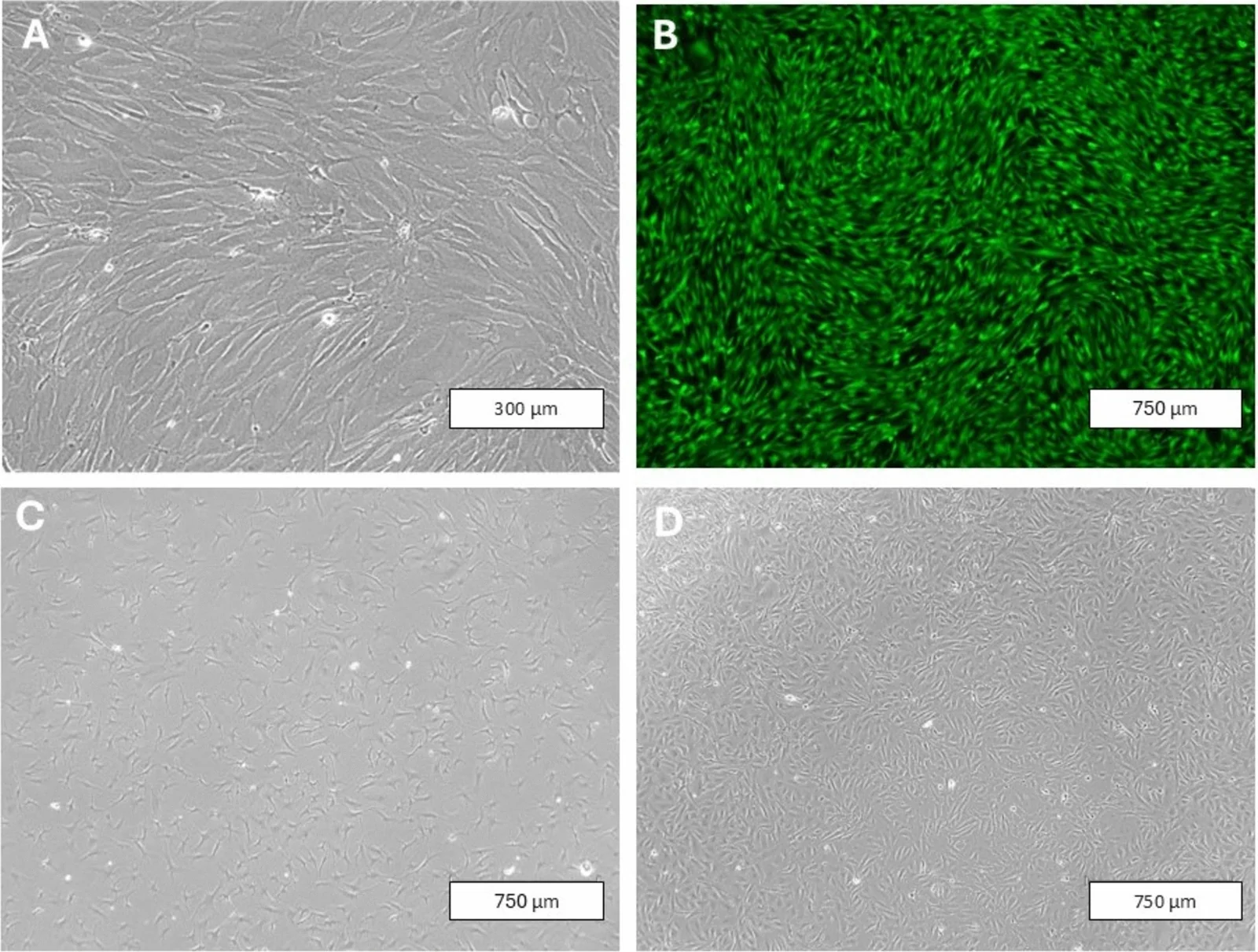
CSFL-1NC cell morphology at different confluency levels. Representative images of CSFL-1NC cell morphology at passage 23 (*A*–*C*) and 40 (*D*). (*A*)Confluent monolayer of CSFL-1NC taken at 20× objective. (*B*) Ca. 90% confluent layer of Calcein-AM stained CSFL-1NC cells taken at 4× objective. (*C*) Sub-confluent layer of CSFL-1NC cells taken at 4× objective. (*D*) CSFL1-NC cells at passage 40 post multiple freeze/thaws. Images taken on an EVOS M5000 microscope.

Within hours of processing and incubation, cells migrating outwards from the explants could be observed. Initially, the cell population appeared heterogeneous in morphology; however, within the first wk, the population began shifting towards a predominantly fibroblastic morphology. The cells were initially maintained in a T25 flask with regular medium changes until confluency was reached, and they were sub-cultured at a ratio of 1:2 and later moved to T75 flasks. Currently, the cell line has been passaged more than 65 times and still retains the same fibroblastic morphology throughout all passages (Fig. 1). Throughout this time, different passages of the cell line have been routinely frozen at −150°C for biobanking purposes. Upon single and multiple freeze/thaws, the cells have retained their morphology and characteristics (Fig. 1). The survival of the cells through multiple freeze/thaw cycles and through > 65 passages split at a 1:2 ratio is indicative of a cell line which has spontaneously immortalised, as is common with fish cell lines, in this case most likely through being in crowded culture over the span of several months before the first passaging (Solhaug *et al*. 2025).

### Proliferation Rate and Growth Conditions

To determine the growth rate and conditions, the cells were grown according to protocol and monitored using the CellTiter-Glo™ Luminescent Cell Viability Assay, and luminescence readings were converted to cell numbers using standard curves, with growth rates and doubling time calculated.

**Figure 2.**
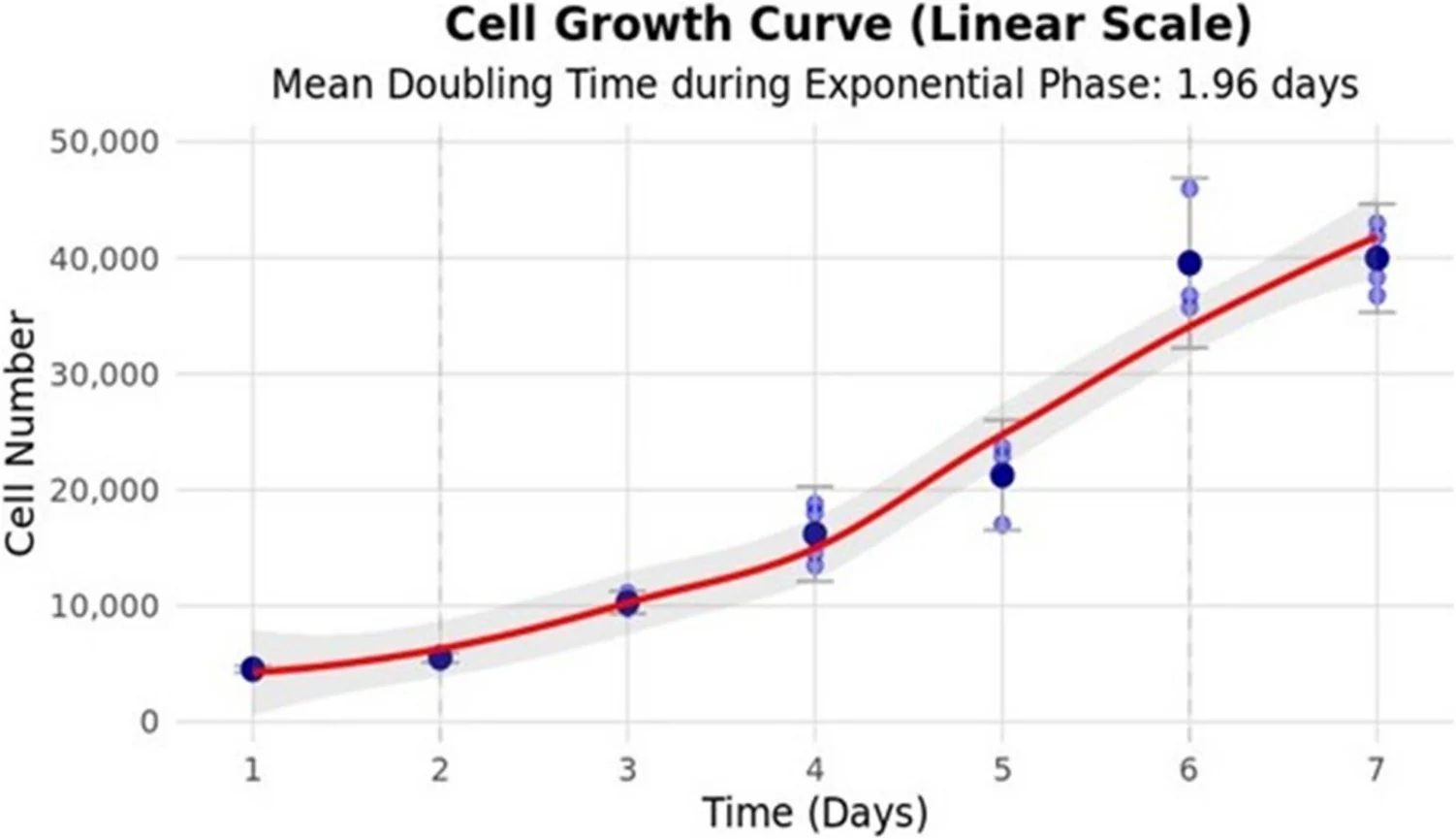
Growth analysis of the CSFL-1NC cell line. The growth of CSFL-1NC cells at passage 15 over the course of 1 wk. Each datapoint represents the mean of 4 replicates, with cell number starting at approximately 5000 cells on day 1 and ending at approximately 40,000 cells at day 7. *Error bars* represent standard deviation from replicate measurements, and *red lines* represent the modelled growth curve with a shaded confidence interval.

As seen in Fig. 2, the cell line has clear and distinct growth characteristics consistent with the different phases expected of cells in culture. Once seeded, the CSFL-1NC cells first undergo a lag phase of approximately 48 h with little to no growth, after which they enter the exponential growth phase. In the course of a 7-d monitoring experiment of the cell growth, this phase progresses from approximately day 2 until day 6. During this time, the cells displayed a mean doubling time of 1.96 d, effectively 2 d, and a maximum growth rate of 0.620 per d. Between days 6 and 7, the cells entered a plateau phase and cell proliferation halted.

### Transcriptomic Analysis of CSFL-1NC

The CSFL-1NC transcriptome has been investigated and analysed based on samples from three passages: 11, 20, and 26. Initial quality control was conducted using star salmon and the nf-core/RNA-seq pipeline (Patel *et al*. 2024).

Across the three passages, 51,004 expressed genes were identified. To keep only genes that were significantly expressed and to reduce background noise, while also keeping depth within the dataset, only genes expressed above 1 TPM were analysed further (see supplementary datasets 3 and 4). This resulted in a new dataset containing 21,833 genes, 42.8% of the original set of genes. This dataset was further filtered to create new datasets containing only the top 1000 genes expressed per passage (see supplementary datasets 5, 6, and 7). These datasets were then used to identify any genes overlapping within the three datasets. In total, 761 genes were found to be present in the top 1000 genes of each of the passages, accounting for an overlap of 60.3% across passages and indicating a degree of transcriptomic stability over time (see supplementary dataset 8).

**Figure 3.**
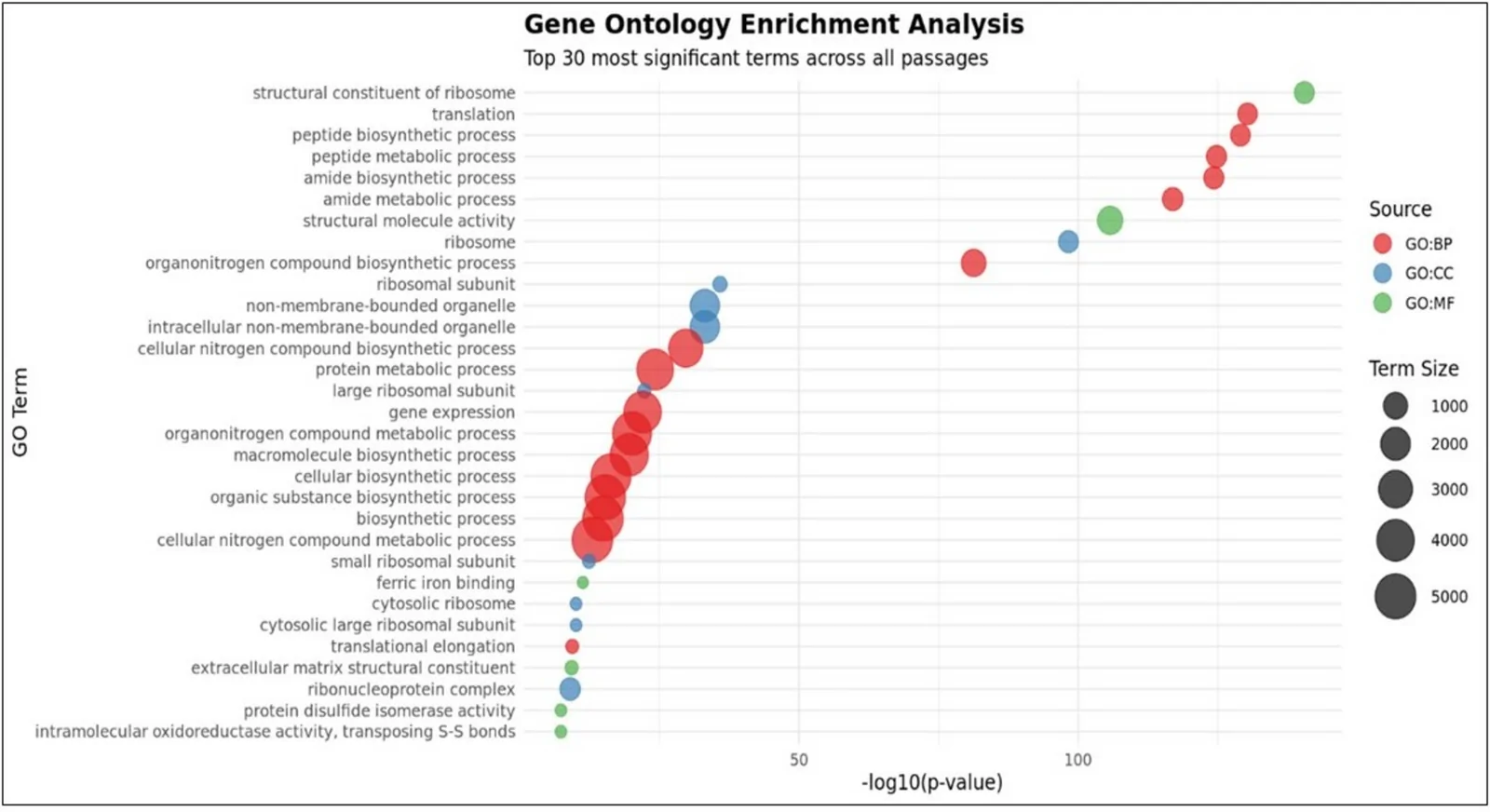
Gene ontology of highly expressed genes. Gene ontology terms identified for the 761 genes in common across the top 1000 genes expressed in each passage. For more details, see supplementary dataset 9.

Next, Gene Ontology (GO) analysis was conducted on the top 761 genes to identify biological processes enriched for the gene set (Fig. 3). Within the 30 most enriched GO terms for this gene set, we found several functional categories indicative of active cellular metabolism. These included translation-related terms ("translation", "structural constituent of ribosome", "ribosomal subunit") and biosynthetic processes ("polypeptide biosynthetic processes", "cellular biosynthetic processes"), suggesting active protein synthesis machinery and metabolic processes involved in cellular growth and maintenance.

**Figure 4.**
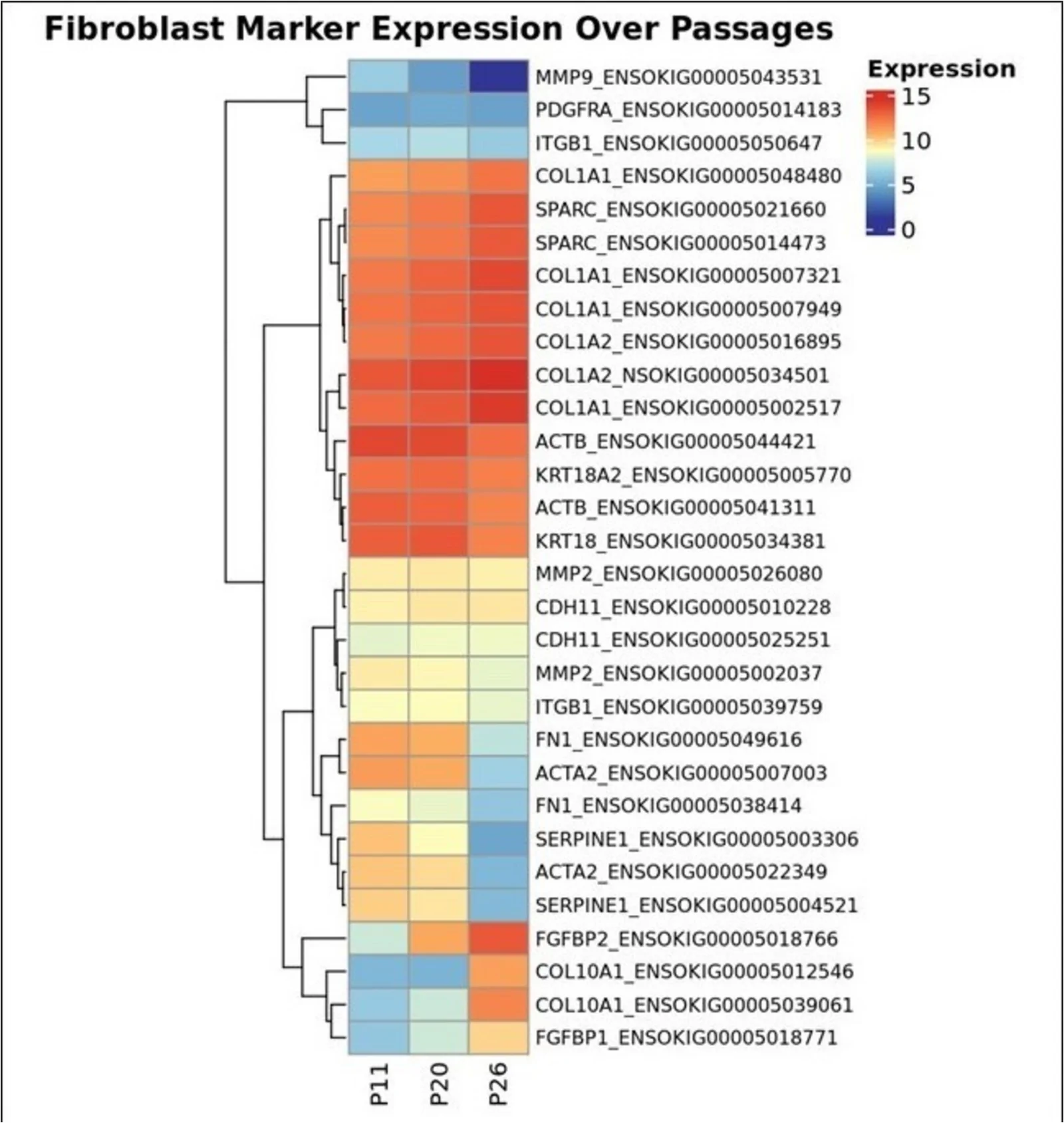
Expression of key fibroblast markers across passages. Heatmap of expression patterns of key fibroblast markers across passages 11, 20, and 26. *Rows* represent individual markers with hierarchical clustering to group markers by similarity in expression. Expression levels are mapped using a log2 scale.

Next, a set of fibroblast markers was identified from existing literature (Sun *et al.* 2016; Muhl *et al*. 2020; Rajan *et al*. 2023). The dataset of all filtered genes was then used to investigate their, as well as any variants, expression across passages as seen in Fig. 4. Here, we find key fibroblast genes (e.g. collagen and *sparc* genes) within extracellular matrix production and organisation within a cluster of genes expressed highly across all passages. Other markers such as *fn1* show somewhat variable expression. *pdgfra* and *tgfb1*, both important in the regulation of fibroblasts, exhibit moderate expression. Both matrix metalloproteinases investigated, *mmp2* and *mmp9*, show lower expression. Interestingly, *vim*, a commonly used marker for fibroblasts, showed low expression across all passages, whereas *krt18*, commonly used as an epithelial cell marker, showed consistently high expression. When further investigating the dataset of the 761 genes in common, we find the same cluster of highly expressed genes seen here within the highest expressed genes, with some genes such as *fn1* and *acta2* only appearing in the list of highest expressed genes for passages 11 and 20, whereas *fgfbp1* only appears in the list of top expressed genes of passage 26. Additionally, a manual search for immune-related genes showed the presence and expression of interleukins such as *il11* and *il34* as well as *cd40*, *slc35a1*, *slc7a5*, and others (see supplementary datasets 3 and 4).

**Figure 5.**
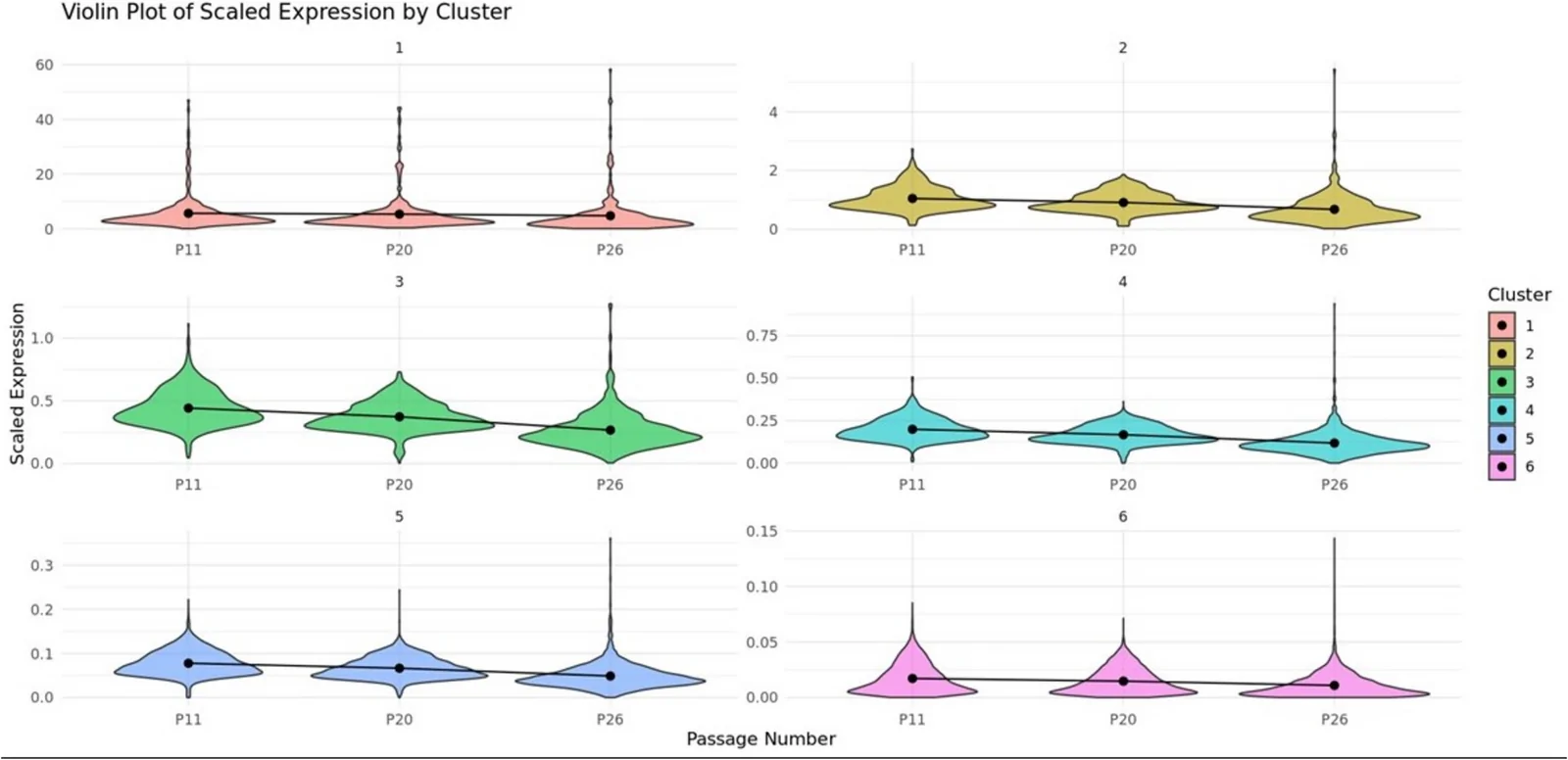
*Violin plot* of scaled gene expression by cluster across passages. Visualisation of scaled gene expression values across passages (P11, P20, P26). Each *panel* represents one cluster, with scaled expression on the *y-axis* and passage number on the *x-axis*. The width of each *violin* represents the density of data points at a given expression level, while *black dots* indicate the median expression within each passage. Note different *y-axis* scales to highlight intra-cluster trends.

To further investigate groupings of genes with similar expression, a clustering analysis was completed using the dataset of all genes above 1 TPM in expression. Six key clusters of genes with similar expression patterns were identified across the three passages, as seen in Fig. 5. The distribution of genes shows that most fall within cluster 6 (*n* = 14,245) and cluster 5 (*n* = 4503) with cluster 1 containing the fewest genes overall (*n* = 259). Within cluster 1, the highest-expressed genes can be found, including several collagen genes and variants, *sparc* variants, and *acta2* variants, all of which were included in the list of key fibroblast markers. In cluster 1, there is a wide distribution in scaled expression; however, this remains the same across all three passages. Cluster 2 shows much lower expression than cluster 1, with a small increase in expression at passage 26. Cluster 3 shows moderate expression levels across all three passages, and cluster 4 shows low but stable expression levels. Clusters 5 and 6 both have low expression values with little to no expression changes across passages. Common to all clusters is a consistent median expression across passages.

### Cell Morphology and Cell Identity

**Figure 6.**
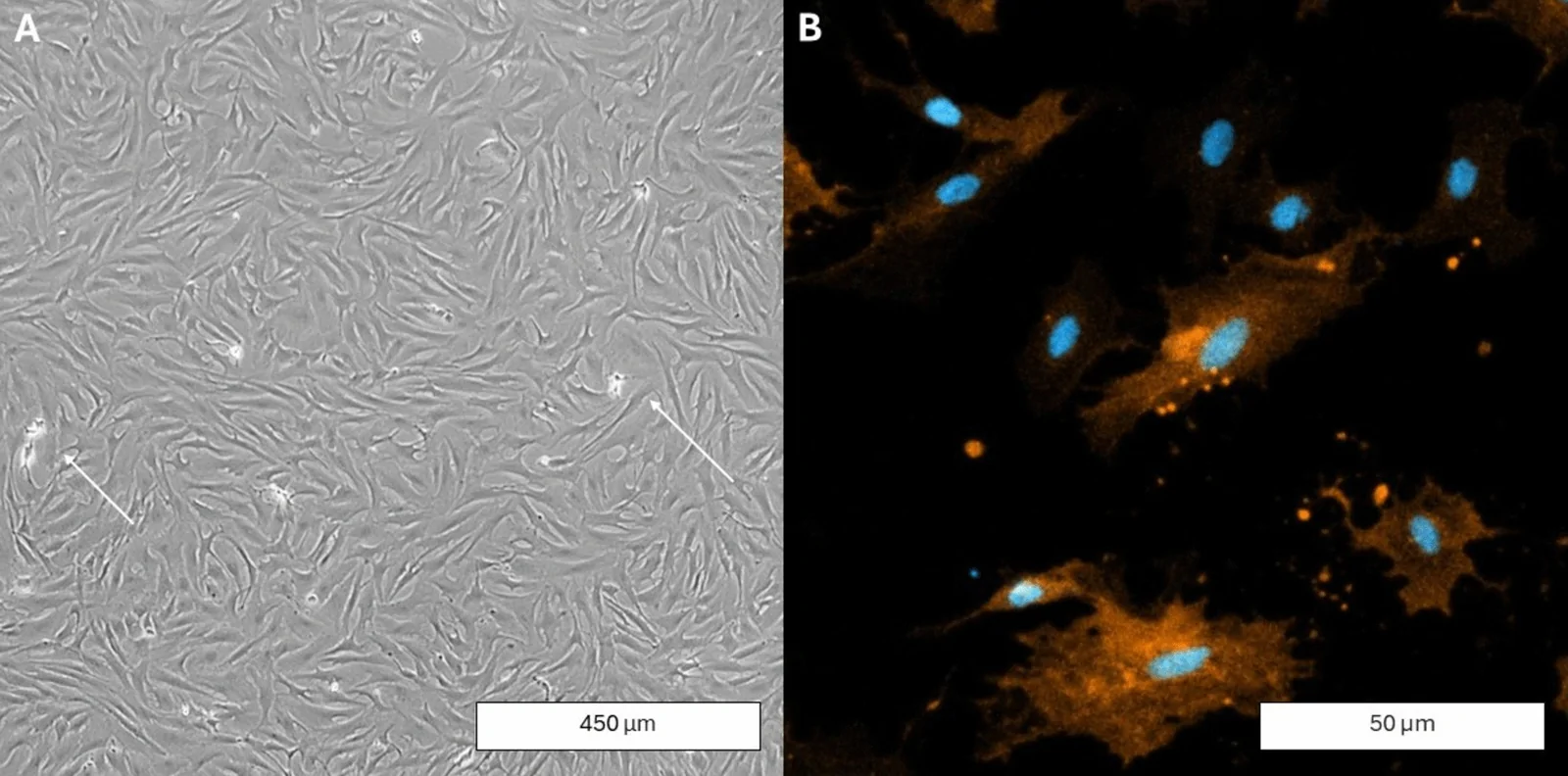
Representative images of CSFL-1NC cells. (*A*) Image taken on EVOS M5000 brightfield microscope using 10× objective. *Arrow* denotes actively dividing cells. Cells are at passage 25. (*B*) Immunostaining α-SMA (*orange*) and DAPI nuclei staining (*blue*) taken at 20× objective. Image taken using confocal microscopy (ZEISS). Cells are at passage 28.

The CSFL-1NC has a homogenous and consistent fibroblastic morphology of long, thin, and spindle-like cells. As seen in Fig. 1*C*, once sub-confluent, the cells will take on a triangular cell shape with multiple extrusions, whereas at full confluency, they will become longer and thinner in shape, as seen in Figs. 1 and 6*A*, both of which are consistent with fibroblastic morphology (Knoedler *et al.* 2023; Parker *et al.* 2023). This morphology has remained stable across all passages since the cell line was initiated, with no major deviations. The homogeneity of the cell line has been confirmed by size-based cell sorting and re-seeding of the different groupings to assess differences in group morphology and development, with none being found (see Supplementary Fig. 1).

To further investigate the suspected fibroblastic cell identity, the cells were stained with an antibody against α-SMA, a genetic marker commonly used to identify activated fibroblasts (Fig. 6*B*) (Nielsen *et al*. 2019). ASG10 cells, a cell line from Atlantic salmon cells and with an epithelial cell identity, were used as a negative control to compare against (see Supplementary Fig. 2) (Gjessing *et al.* 2018). As seen in Fig. 3*D*, the CSFL-1NC cell line shows positive staining against α-SMA, with some variation potentially due to cell cycle or differentiation stages, indicating a fibroblastic morphology.

### Cell Migration

To test the migratory capabilities of the CSFL-1NC cell line, a wound healing assay was conducted using the creation of an artificial wound/scratch. To ensure cells would not proliferate into the open space of the induced wound but would instead migrate, they were given growth medium containing only 2% FBS to facilitate migration. The cells were imaged daily to assess whether any migration had occurred.

**Figure 7.**
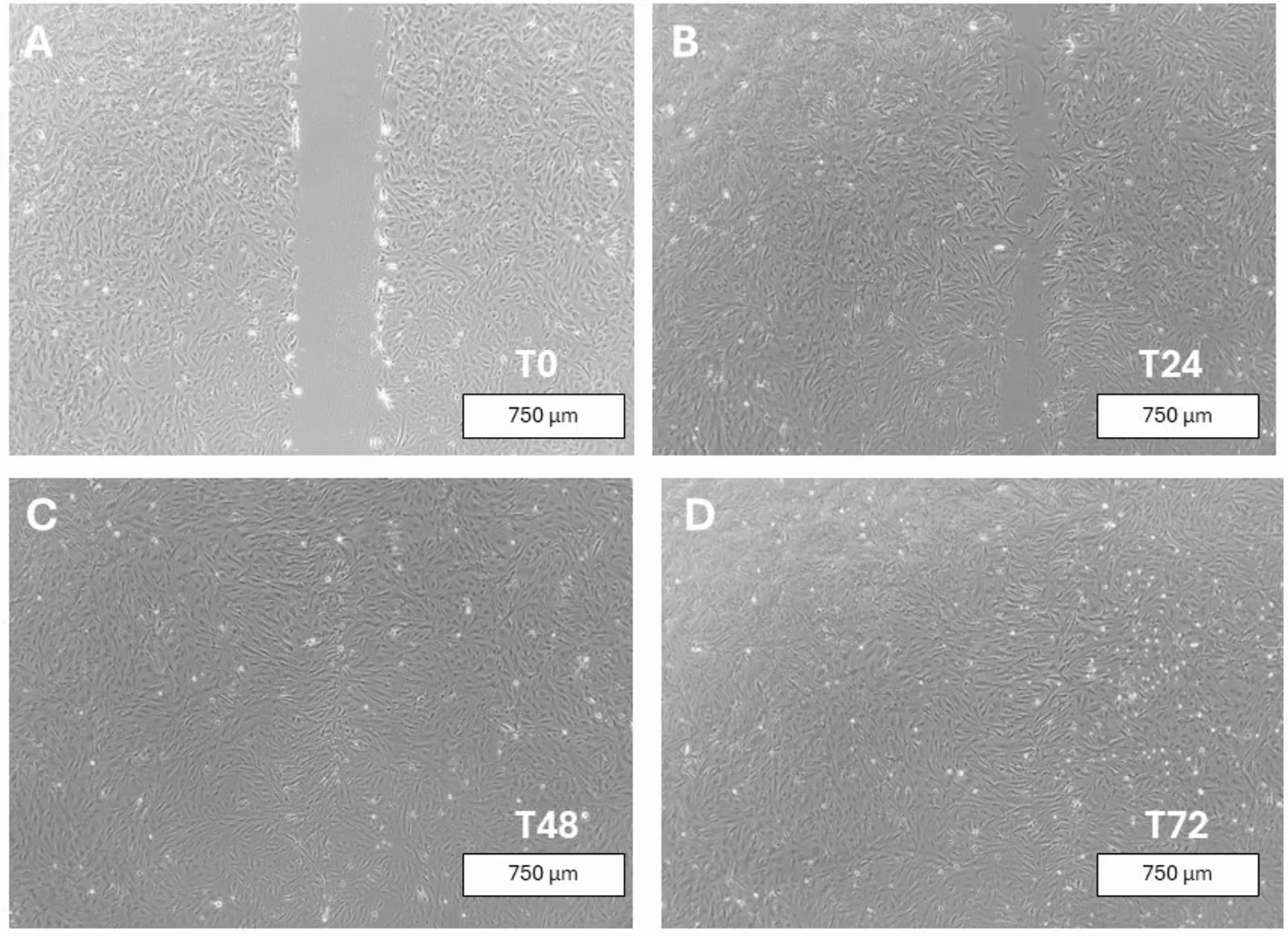
Wound healing assay of CSFL-1NC cells. Representative scratch assay of the CSFL-1NC cell line at passage 18. (*A*) Timepoint 0 where the wound had been created right before imaging. (*B*) 24 h post wounding. (*C*) 48 h post wounding. (*D*) 72 h post wounding. All images taken on an EVOS 5000 microscope system with a 4× objective.

Within 24 h post creation of the artificial wound, the cells had already markedly lessened the open space of the induced wound. The cells migrating from one edge to another of the wound appear elongated in shape, with some stretching almost all the way across the wound and onto the other side. At 48 h post wounding, the cells now fully occupied the open space created by the wound and appear almost confluent. However, as seen in Fig. 7*D*, the cells now occupying the space where the wound was now appeared somewhat different in morphology of growth pattern compared to the other cells. They appear to grow more “sideways” next to one another, likely to most effectively close the gap between cells, making it easy to spot where the wound once was. The same can be seen in Fig. 7*C* at 72 h post wounding/scratching, where there is no open space left.

### Viral susceptibility

The CSFL-1NC cells were exposed to ISAV and IPNV to determine if the cells were permissible to infection. Cells were inoculated with two different viral loads of 0.1 or 0.5 MOI and analysed through imaging and qPCR using RNA extracted from the cells. The CSFL-1NC cells were permissible to infection with IPNV as clear cytopathic effects were seen within 5 d (see supplementary data, Fig. 3) at both 0.1 and 0.5 MOI. No such effects were observed in the cell population infected with ISAV.

**Figure 8.**
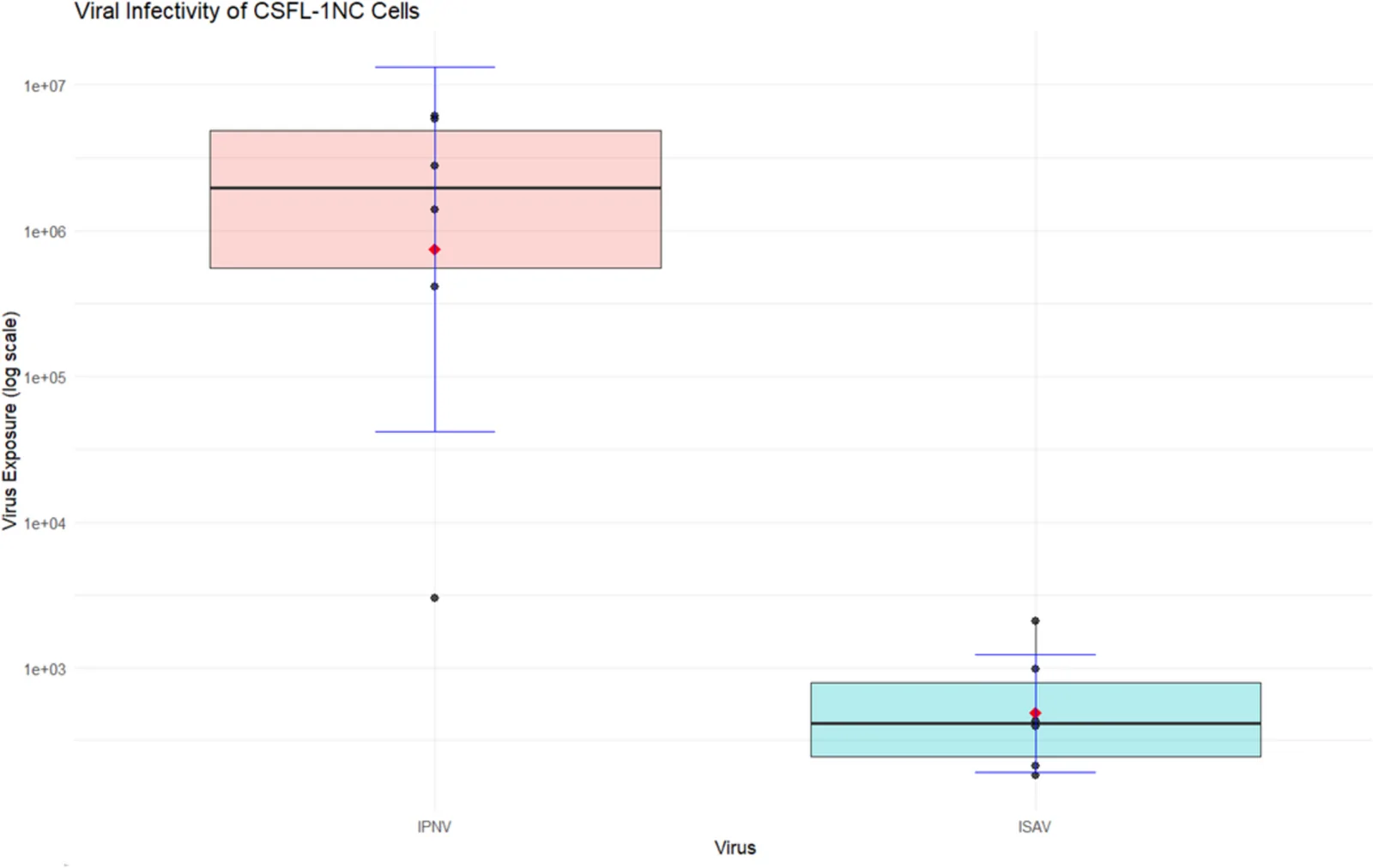
Viral infectivity of CSFL-1NC cells. *Box plot* showing log_10_-transformed fold change (*y-axis*) in viral RNA relative to the reference gene and control samples for IPNV and ISAV. The median is shown as the *centre line*, the interquartile range as the *box*, and full data range (excluding outliers) as *whiskers*. Individual data points are shown as *dots*, *red diamonds* indicate group means, and *blue error bars* indicate ± 1 standard deviation.

Further, we wanted to see if we could detect virus RNA in the cells, indicating that the virus had entered the cells. Virus RNA from cells exposed to 0.5 MOI of IPNV or ISAV was measured with qPCR, estimating relative RNA level normalised to a reference gene and compared to control samples. In control samples, the viral amplicons were not amplified (Ct = 40). Our qPCR data showed a high degree of variation, but the results gave a clear indication of the difference in infectivity between the two viruses. Samples exposed to IPNV showed high levels of viral RNA at a level of 2.7 × 10^6^ ± 2.7 × 10^6^ (mean ± SD; normalised to the reference gene), indicating an almost 3-million-fold increase in virus RNA level relative to the control samples. The cells exposed to ISAV showed much lower viral load, having a relative ISAV RNA level of 194 ± 124-fold change compared to the control samples (Fig. 8).

### Transfection and transduction

**Figure 9.**
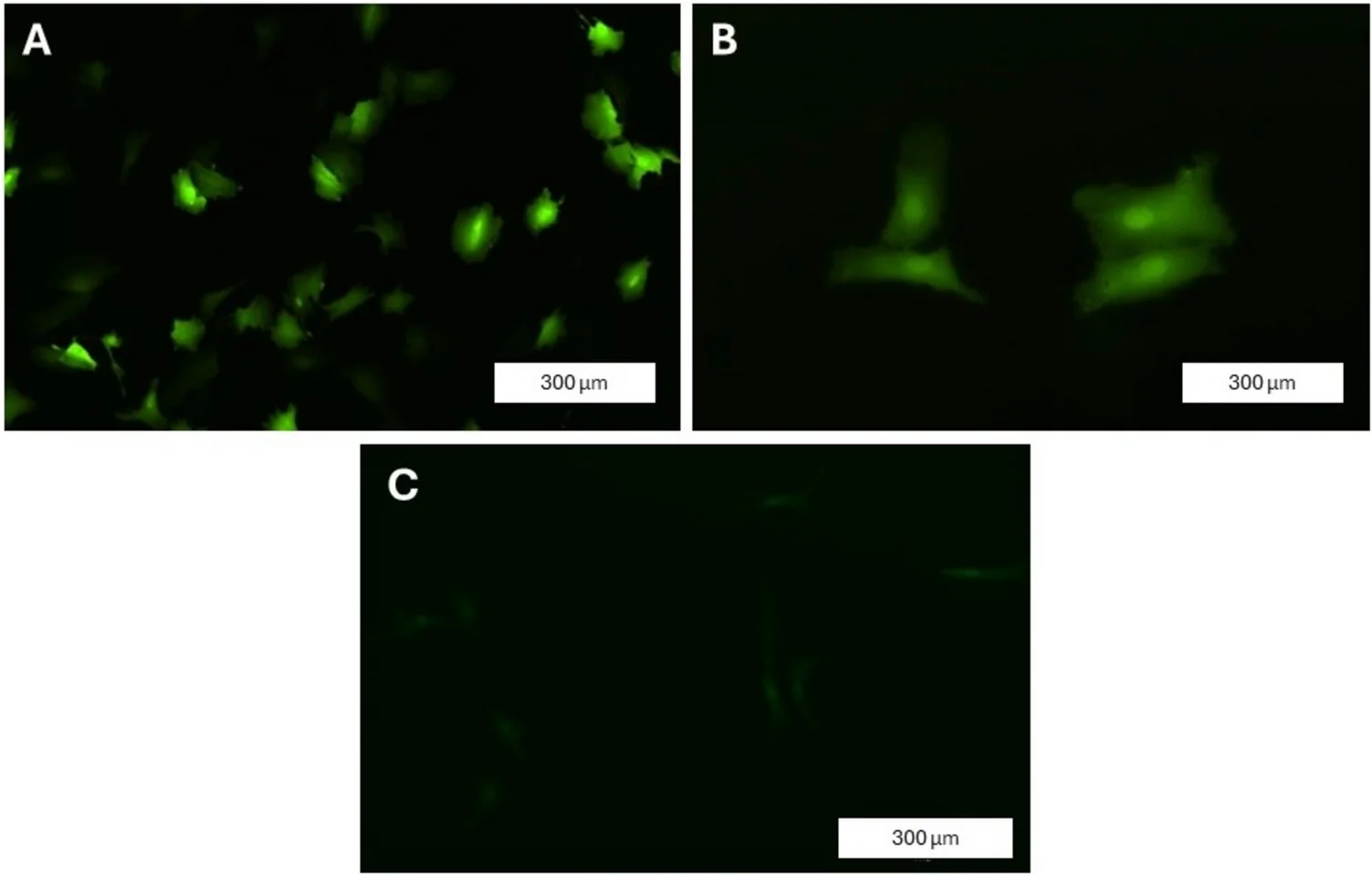
Positive GFP CSFL-1NC cells from transfection and transduction. Representative images showing CSFL-1NC cells positive for GFP post transfection or transduction. (*A*) Cells 24 h post transfection. Cells show a strong signal but some abnormal cell morphology. *Scale bar* = 100 μm. (*B*) Cells 1 wk post transfection showing a strong signal and regular cell morphology. *Scale bar* = 50 μm. (*C*) Cells 5 d post lentiviral transduction. Cells show normal morphology but weaker positive signalling*. Scale bar* = 300 μm. Transfected cells are at passage 30 and transduced at passage 38. All images taken with an Olympus microscope at 10× (*A*, *C*) and 20× (*B*) magnification.

Using a GFP reporter plasmid for easy visualisation, the CSFL-1NC cell line was both transfected and transduced successfully, however with better efficiency and clearer staining once transfected then transduced as seen in Fig. 8. Cells transfected had somewhat of an irregular shape immediately post transfection; however, they were positive for GFP within 24 h and showed a much more homogeneous morphology 1 wk post transfection, where they returned to a more classic fibroblast morphology. As seen in Fig. 9, cells transduced with a lentiviral system did not show the same efficiency as the transfected cells and required higher magnification to best visualise cells. While overall initial cell health seems initially better post transduction, the efficiency was relatively low, with no GFP seen until days 4–5.

### Gene editing

**Figure 10.**
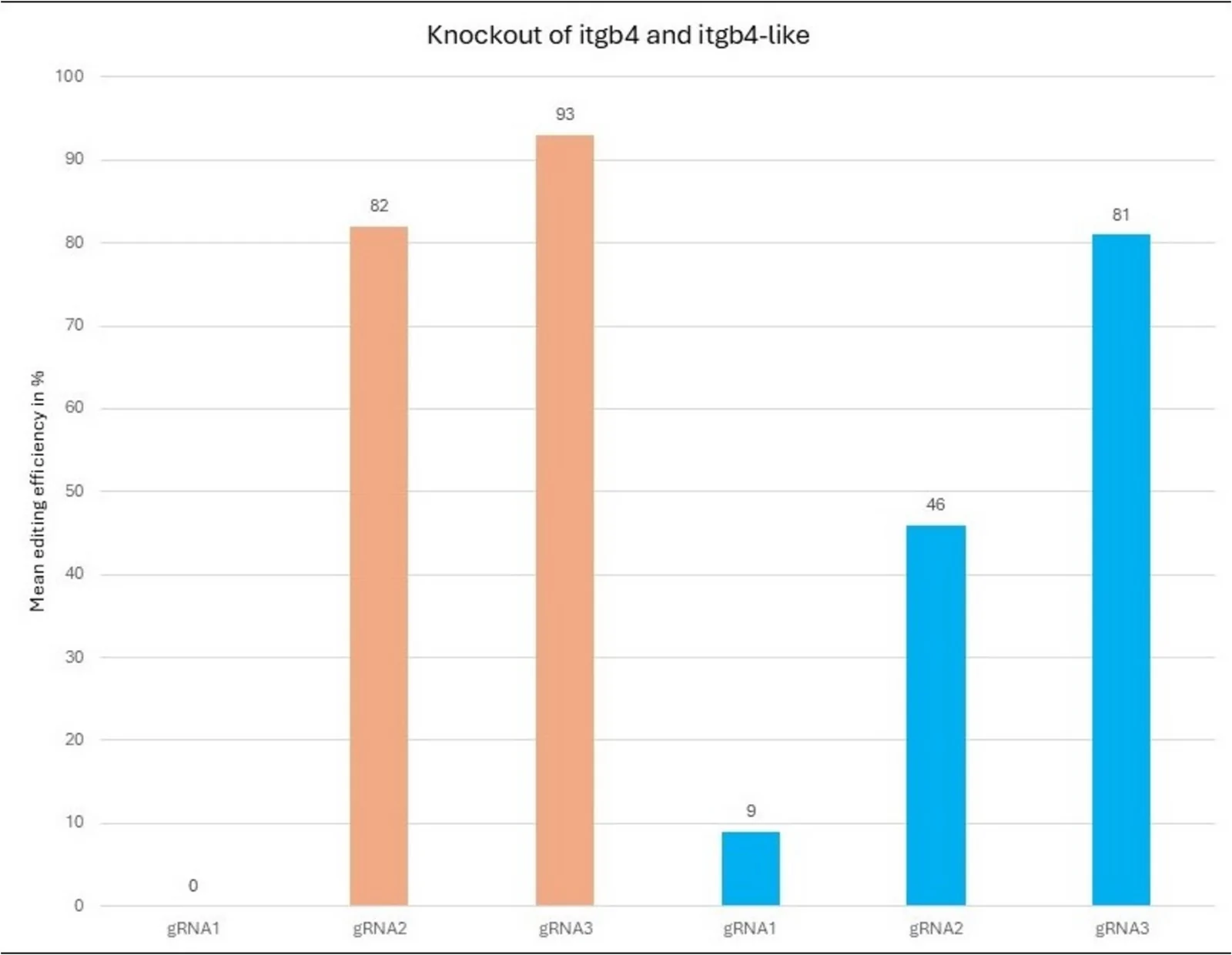
CRISPR knock out efficiency in CSFL-1NC. *Graph* showing the mean editing (KO) efficiency using 6 different gRNAs and targeting two genes: *itgb*4 (*orange*) and *itgb4-like* (*blue*) in CSFL-1NC cells at passage 31. Each percentage represents a mean generated from 3 replicates.

To assess the permissiveness of the cell line to gene editing via CRISPR-Cas9, six different guides targeting the *itgb4* gene and *itgb4-like* gene were designed and tested, with three guides targeting *itgb4* and three targeting *itgb4-like* (Fig. 10). CRISPR components in the form of ribonucleoprotein complex (RNPs), consisting of Cas9 and the gRNAs, were delivered to the cells through electroporation. A successful knock out of the genes was determined by estimating editing efficiency post-PCR and sequencing of edited fragments. All six gRNAs were tested separately, and cells were sampled 1 wk post-transfection. Cells did not show any morphological changes post-editing. Out of the six gRNAs, gRNA3 designed for *itgb4* and gRNA3 designed for *itgb4-like* showed the highest editing efficiency with 93% and 81% indel efficiency, respectively. Both gRNA2s showed moderate efficiency (82% and 46%, respectively) with gRNA1 targeting *itgb4*-like showing the lowest efficiency rate of only 9%. gRNA1 targeting *itgb4* did not show any edits and can be assumed not to work. Overall, the editing efficiency was markedly higher in the gRNAs targeting *itgb4* as opposed to *itgb4-like* with the exception of both gRNA1s.

## Discussion

Here, we have presented the first cell line from an adult coho specimen, as well as some preliminary characterisation of the cell line. Hopefully, the cell line will prove to be a useful tool for the in vitro studies of coho salmon innate immunity in relation to common salmonid pathogens, as well as in furthering our understanding of fundamental salmonid biology, for example in cross-species comparative studies to investigate species-specific cellular responses and mechanisms.

Throughout 2 yr, this cell line has been maintained with ease in culture through regular medium changes, passaging, and freeze/thawing. Although it initially appeared heterogeneous in morphology, within the first weeks of culture, a predominantly fibroblastic phenotype became dominant. Both comprehensive transcriptomic analyses of common fibroblastic marker genes and positive staining with α-SMA seem to support a fibroblast or fibroblast-like cell identity.

Despite most cell markers originally being characterised in human systems, they do appear to be well enough conserved in fish species to be usable for cell type identification (Rajan *et al*. 2023). We selected key marker genes such as collagen genes, matrix metalloproteinases, fibroblast growth factors, and fibronectin based on existing literature to investigate a relative variety of genes and have in turn discovered distinct expression patterns across passages (Vo *et al*. 2015, 2019; Lynch & Watt 2018; Muhl *et al.* 2020; LeBleu & Neilson 2020). All collagen variants investigated showed a consistent and high expression at all passages, with the exception of *col10a1* variants. Interestingly, *col10a1* variants appear upregulated in passage 26 in comparison to passage 11. As collagen genes are major components of the ECM, where they both change and remodel it, the higher expression of *col10a1* at later passages, in conjunction with the continued high expression of *col1a1* and *col1a2*, could be indicative of an active extracellular matrix adapting to culture conditions (Liang *et al.* 2020). This fits well with the high expression seen of *sparc* variants across passages, as they are important for extracellular matrix synthesis and changes to cell shape (Ham *et al*. 2023). Furthermore, a slight decrease in genes such as *fn1*, *acta2*, and *serpine1* might indicate adaptation to culture conditions rather than a loss of fibroblastic cell identity, as these fibroblasts in native tissue constantly have to respond to mechanical forces and stress they are not exposed to in culture (Fini & Gitard 1990). While some commonly used markers appear to be expressed at negligible levels (e.g. *vim* and *itgb1*), this does not provide strong evidence for discounting a fibroblastic cell identity overall, as fibroblasts are ubiquitous and heterogeneous, with gene expression often dependent on both tissue and species of origin (Ingerslev *et al.* 2010; Plikus *et al*. 2021; Knoedler *et al*. 2023; Parker *et al.* 2023). In fact, the low expression of *vim*, coupled with the high expression of some keratins (e.g. *krt18*) seen here, would be highly unusual in fibroblast cells of mammalian origin; however, this expression pattern has been seen in fish fibroblasts previously and, as such, is not entirely unexpected (Humagain *et al*. 2024).

It is not unusual for cell lines to undergo changes both phenotypically and transcriptomically over time, especially over longer periods in culture (Kong *et al.* 2011; Geraghty *et al.* 2014). Indeed, many cell lines have been shown to upregulate genes in relation to cell proliferation and cell cycle maintenance while downregulating genes modulating contact inhibition. Within the CSFL-1NC transcriptome, we see some parallels to this with fibroblast growth factors (*fbp1* and *fgbp2*) becoming gradually more expressed across passages, likely indicating adaptive changes to maintain growth and survival in culture conditions. It is also imperative to note that the characterisations done in this study pertain to the passages used, which are considered early- to mid-stage passages. However, since salmonid cells are considered immortalised after 20–30 sub-cultivations and successful cryopreservation, we do not expect the cell line to change drastically at later passages (Solhaug *et al*. 2025). Indeed, the CSFL1-NC cell line has exhibited consistent morphology, growth behaviour, and marker expression over a long time, as shown here. Nevertheless, until validated at later passages (Solhaug *et al*. 2025) (e.g. > P50), our data should be considered preliminary characterisations. To fully validate the characteristics of the cell line and ensure it displays long-term stability and robustness, continued monitoring and repletion of characterisation experiments at later passages, such as further RNA sequencing, will be crucial to ascertain the cell line’s overall properties and reliability.

Furthermore, we see a decrease in the expression of both *mmp2* and *mmp9* across passages, suggesting decreased expression of contact inhibition-related genes (Fini & Gitard 1990; Kong *et al.* 2011). Nevertheless, the transcriptome of CSFL-1NC appears to be relatively stable, with 761 genes shared amongst the top 1000 genes of each passage, accounting for more than 60%. Furthermore, the GO terms enriched for CSFL-1NC cells from all passages, including all genes expressed above the 1 TPM threshold, show enrichment of terms associated with fibroblastic cell behaviour (e.g. “extracellular matrix structural constituent” as well as many terms indicative of active cells making new proteins and carrying out various life-essential cellular functions (e.g. “translation”, “peptide biosynthetic processes”, “cellular biosynthetic processes”).

In order to assess whether CSFL-1NC could be used as an in vitro tool to study viral resistance/susceptibility in coho salmon, it was challenged with both IPNV and ISAV. IPNV poses significant threats to both aquaculture and wild populations, while interestingly, coho salmon do both get infected and carry ISAV, yet only display significant negative pathologies in the presence of other factors such as other secondary infections or general poor health (Kibenge *et al*. 2001; Jorquera *et al*. 2016; Alarcon *et al*. 2020; Mordecai* et al*. 2021). The cytopathic effects induced by IPNV in CSFL-1NC, even at 0.1 MOI, indicate clear susceptibility with lethal effects, supported by qPCR data showing high levels of IPNV RNA in the infected cells compared to control samples. Interestingly, the CSFL-1NC cells did not show any cytopathic effects once infected with ISAV at either MOI. The virus RNA was detectable in the infected samples, albeit at a much lower level than the IPNV. While these are only preliminary results, in need of both further investigation and validation, they might be indicative of the cell line retaining the response of coho salmon in vivo, as we see little to no response. This differential susceptibility observed both in vivo and in vitro might be indicative of species-specific immune mechanisms that warrant further investigation. The CSFL-1NC cell line could perhaps, in conjunction with other cell lines from species more susceptible to ISAV, be used to investigate underlying genetic components responsible for viral tolerance and perhaps be used in functional studies to assess the importance of specific genes or pathways in conferring resistance.

Finally, the CSFL-1NC cell line has been proven to be amenable to several methods of gene editing, certainly making a case for its future usage. High efficiency in CRISPR KOs and high efficiency when transfected suggest potential for future studies using more advanced methods. While transduction efficacy was low, this is not very surprising, as lentiviral transduction of salmonid cell lines has only recently become fully streamlined, with several salmonid cell lines still being notoriously difficult to transduce efficiently (Gratacap *et al.* 2020). As such, while currently ineffective, these results lay the groundwork for future editing of the cell lines using lentiviral transduction.

## Supplementary Information

Below is the link to the electronic supplementary material.

Supplementary Figure 1. Size Based Flow Cytometry of CSFL-1NC Cells. Size based sorting of CSFL-1NC cells from cell sorting showing all events. (*A*) Area in which most cells cluster. (*B*) Outliers from group A. (JPG 75.3 KB)

**Supplementary Figure 2.**
Immunostaining of ASG10 and CSFL-1NC Cells. (*A*) ASG10 cells at passage 32 showing only staining of nuclei (blue) and no signal detected α-SMA (orange). (*B*) CSFL-1NC at passage 28 stained for a-SMA (orange) and DAPI nuclei staining (blue). Images taken using confocal microscopy (ZEISS) fitted with a 20X objective. (JPG 59.3 KB)

Supplementary Figure 3. Viral Infection CSFL-1NC. Pictures taken of viral infection challenge at day 5 (cells at passage 36). (*A* & *D*) Negative controls of cells grown in growth medium with 2% FBS. (*B*) Cells infected with ISAV at MOI 0.1. (*C*) Cells infected with ISAV at MOI 0.5. (E) Cells infected with IPNV at 0.1 MOI. (*F*) Cells infected with IPNV at MOI 0.5 Cells infected with IPNV show clear cytopathic effects. (JPG 118 KB)

Supplementary Dataset file 1 (XLSX 9.97 KB)

Supplementary Dataset file 2 (XLSX 10.4 KB)

Supplementary Dataset file 3 (XLSX 3.62 MB)

Supplementary Dataset file 4 (XLSX 1.95 MB)

Supplementary Dataset file 5 (XLSX 86.6 KB)

Supplementary Dataset file 6 (XLSX 87.1 KB)

Supplementary Dataset file 7 (XLSX 87.8 KB)

Supplementary Dataset file 8 (XLSX 88.1 KB)

Supplementary Dataset file 9 (XLSX 30.5 KB)

Supplementary Dataset file 10 (XLSX 279 KB)

Supplementary Dataset file 11 (XLSX 1.02 MB)

## Data Availability

Sequencing data has been deposited in the European Nucleotide Archive (ENA) under accession number PRJEB90578.
